# Transmission Characteristics and Inactivated Vaccine Effectiveness Against Transmission of SARS-CoV-2 Omicron BA.5 Variants in Urumqi, China

**DOI:** 10.1001/jamanetworkopen.2023.5755

**Published:** 2023-03-30

**Authors:** Kai Wang, Zihao Guo, Ting Zeng, Shengzhi Sun, Yanmei Lu, Jun Wang, Shulin Li, Zemin Luan, Huling Li, Jing Zhang, Yida Wang, Yaoqin Lu, Shi Zhao

**Affiliations:** 1 State Key Laboratory of Pathogenesis, Prevention, and Treatment of High Incidence Diseases in Central Asia, Department of Medical Engineering and Technology, Xinjiang Medical University, Urumqi, China; 2 Jockey Club School of Public Health and Primary Care, Chinese University of Hong Kong, Hong Kong, China; 3 School of Public Health, Xinjiang Medical University, Urumqi, China; 4 Department of Epidemiology and Biostatistics, School of Public Health, Capital Medical University, Beijing, China; 5 Department of Cardiac Pacing and Electrophysiology, First Affiliated Hospital of Xinjiang Medical University, Urumqi, China; 6 Urumqi Center for Disease Control and Prevention, Urumqi, China; 7 Centre for Health Systems and Policy Research, Chinese University of Hong Kong, Hong Kong, China

## Abstract

**Importance:**

In 2022, Omicron variants circulated globally, and Urumqi, China, experienced a COVID-19 outbreak seeded by Omicron BA.5 variants, resulting in the highest number of infections in the city’s record before the exit of the zero COVID-19 strategy. Little was known about the characteristics of Omicron variants in mainland China.

**Objective:**

To evaluate transmission characteristics of Omicron BA.5 variants and the effectiveness of inactivated vaccine (mainly BBIBP-CorV) against their transmission.

**Design, Setting, and Participants:**

This cohort study was conducted using data from an Omicron-seeded COVID-19 outbreak in Urumqi from August 7 to September 7, 2022. Participants included all individuals with confirmed SARS-CoV-2 infections and their close contacts identified between August 7 and September 7, 2022 in Urumqi.

**Exposures:**

A booster dose was compared vs 2 doses (reference level) of inactivated vaccine and risk factors were evaluated.

**Main Outcomes and Measures:**

Demographic characteristics, timeline records from exposure to laboratory testing outcomes, contact tracing history, and contact setting were obtained. The mean and variance of the key time-to-event intervals of transmission were estimated for individuals with known information. Transmission risks and contact patterns were assessed under different disease-control measures and in different contact settings. The effectiveness of inactivated vaccine against the transmission of Omicron BA.5 was estimated using multivariate logistic regression models.

**Results:**

Among 1139 individuals diagnosed with COVID-19 (630 females [55.3%]; mean [SD] age, 37.4 [19.9] years) and 51 323 close contacts who tested negative for COVID-19 (26 299 females [51.2%]; mean [SD] age, 38.4 [16.0] years), the means of generation interval, viral shedding period, and incubation period were estimated at 2.8 days (95% credible interval [CrI], 2.4-3.5 days), 6.7 days (95% CrI, 6.4-7.1 days), and 5.7 days (95% CrI, 4.8-6.6 days), respectively. Despite contact tracing, intensive control measures, and high vaccine coverage (980 individuals with infections [86.0%] received ≥2 doses of vaccine), high transmission risks were found in household settings (secondary attack rate, 14.7%; 95% CrI, 13.0%-16.5%) and younger (aged 0-15 years; secondary attack rate, 2.5%; 95% CrI, 1.9%-3.1%) and older age (aged >65 years; secondary attack rate, 2.2%; 95% CrI, 1.5%-3.0%) groups. Vaccine effectiveness against BA.5 variant transmission for the booster-dose vs 2 doses was 28.9% (95% CrI, 7.7%-45.2%) and 48.5% (95% CrI, 23.9%-61.4%) for 15-90 days after booster dose. No protective outcome was detected beyond 90 days after the booster dose.

**Conclusions and Relevance:**

This cohort study revealed key transmission characteristics of SARS-CoV-2 as they evolved, as well as vaccine effectiveness against variants. These findings suggest the importance of continuously evaluating vaccine effectiveness against emerging SARS-CoV-2 variants.

## Key Points

**Question:**

What were the transmission characteristics of SARS-CoV-2 Omicron BA.5 variants, and was inactivated vaccine associated with a protective outcome against the transmission of these variants?

**Findings:**

This cohort study of 1139 individuals with COVID-19 found that despite active contact tracing, high vaccine coverage, and other intensive control measures, Omicron BA.5 variants had high risks of transmission in household settings and among younger and older individuals. Compared with a 2-dose inactivated vaccine, a booster dose was associated with a protective outcome against Omicron BA.5 transmission.

**Meaning:**

These findings suggest that there was high transmission risk of Omicron BA.5 variants but the combination of vaccine and nonpharmaceutical interventions may be associated with reduced transmission.

## Teaser

This cohort study investigates transmission characteristics and vaccine effectiveness against transmission of SARS-CoV-2 Omicron BA.5 variants in China.

## Introduction

As of September 26, 2022, the COVID-19 pandemic caused by SARS-CoV-2 had led to more than 611 million infections and 6.5 million deaths globally.^1^ Owing to the continual evolution and adaptation of the virus, variants of SARS-CoV-2 with greater transmissibility and immune escape have persistently emerged,^2,3^ posing significant challenges for mitigation through public health and social measures (PHSMs) and vaccination.

SARS-CoV-2 Omicron variants, which were identified in South Africa in late 2021, have been designated by the World Health Organization as the fifth variant of concern and have dominated the pandemic. Initial Omicron outbreaks across the world were caused by the BA.1 lineage. Since early 2022, BA.1 has been quickly replaced by the Omicron BA.2 sublineage, which has greater transmissibility.^4^ Omicron BA.4 and BA.5 variants were the sublineages of BA.2; owing to their substantial resistance to antibodies elicited by vaccination or by infection with Omicron BA.1 or BA.2 variants,^5^ Omicron BA.4 and BA.5 variants have replaced Omicron BA.2 variants and recently caused major outbreaks globally.^6^ As of September 19, 2022, Omicron BA.5 variants and descendants have become the dominant circulating strain, accounting for 76.6% of globally sequenced infections, followed by the Omicron BA.4 variant and its descendants, with 7.5%.^7^

Understanding the epidemiological characteristics of Omicron BA.5 variants is crucial for planning control strategies for future outbreaks. In most countries, fundamental PHSMs, such as contact tracing, isolation, and quarantine of individuals who were infected and their contacts, have been key for mitigating outbreaks. Knowledge of the distributions of time intervals between key events of transmission may provide insights into the temporal aspects of these PHSMs. For instance, the incubation period, defined as the time between infection and clinical onset, could help to define the duration of isolation and quarantine periods for close contacts.^8^ The generation interval (GI), defined as the time between secondary and primary infections, may inform the responsiveness of contact-tracing measures.^9^ The viral shedding period may help determine the length of isolation for individuals with asymptomatic infections. We searched the PubMed database for articles about transmission characteristics of Omicron BA.5 published from January 1 to October 20, 2022, and found 1 peer-reviewed observational study evaluating transmission risks of Omicron BA.5 variants their key epidemiological parameters, which was based on data from South Africa.^10^ Transmission characteristics of Omicron BA.5 were largely unassessed for other locations, especially for regions with a background of COVID-19 elimination policies. It is thus important to monitor these key epidemiological parameters for emerging genetic variants of SARS-CoV-2 given that they vary across variants.^3,11^

Although Omicron BA.5 variants had a greater immune escaping ability than BA.2, there is little and conflicting evidence regarding vaccine effectiveness (VE) against Omicron BA.5 variants. A 2022 study^12^ found decreased VE against hospitalization and death for Omicron BA.5 variants compared with Omicron BA.2 variants, whereas studies from South Africa^13^ and the UK^14^ found no evidence of reduced VE against hospitalization for BA.5 variants compared with BA.2 variants. In addition, there is a lack of studies assessing VE against transmission,^15^ which could implicitly answer questions about the extent to which vaccines are associated with decreased risk of onward transmission from vaccinated individuals with COVID-19. For the protective outcome associated with vaccines, a 2022 systematic review^16^ identified 3 peer-reviewed studies published before March 8, 2022, that investigated VE against the transmission of Omicron variants; 1 of these observational studies adjusted for confounding variables when estimating vaccine effectiveness,^4^ and all studies analyzed Omicron BA.1 or BA.2 variants. We searched the PubMed database for studies in clinical practice of VE against Omicron transmission published from March 1 to October 20, 2022, but found no peer-reviewed observational studies on the topic. As of October 2022, to our knowledge, no estimates of the effectiveness of inactivated vaccine against Omicron BA.5 variants were reported. Owning to the previous zero COVID-19 policy, SARS-CoV-2 was at a relatively low level in China compared with most of the rest world, and thus no previous estimates of the effectiveness of COVID-19 vaccines against the transmission of SARS-CoV-2 Omicron variants in China were published before December 2022.

In this study, we analyzed a comprehensive set of contact-tracing data collected during outbreaks seeded by SARS-CoV-2 Omicron BA.5.2 variants in Urumqi, China. We estimated epidemiological features, infectivity, and VE against transmission for the Omicron BA.5.2 sublineage.

## Methods

This was a retrospective cohort study including all individuals with laboratory-confirmed SARS-CoV-2 infections and their close contacts identified from August 7 to September 7, 2022 in Urumqi, China. This study was reported following the Strengthening the Reporting of Observational Studies in Epidemiology (STROBE) reporting guideline. The collection of specimens and epidemiological and clinical data for individuals infected with SARS-CoV-2 and their close contacts was a part of a continuing public health investigation of COVID-19 outbreaks, which was determined in the Protocol on the Prevention and Control of COVID-19 by the National Health Commission of the People’s Republic of China to be exempt from ethical approval (ie, institutional review board assessment). All study data were completely anonymized. This study was approved by the institutional ethics committee of Xinjiang Medical University. Because this study was a retrospective analysis using secondary data without personal identity or human samples, the requirement for obtaining informed consent was waived by the Urumqi Center for Disease Control and Prevention.

### Study Setting and Design

Before August 2022, no large-scale COVID-19 outbreak occurred under the context of zero COVID-19 control measures in Urumqi. As of July 2022, vaccine coverage was 90% among the general population in mainland China for the 2-dose inactivated vaccine (mainly BBIBP-CorV [Sinopharm]); more than 72% of this population had already received the third dose.

The first confirmed SARS-CoV-2 infections seeded by the SARS-CoV-2 Omicron BA.5.2 variant were reported on August 7, 2022, in Urumqi. This variant spread rapidly in the population, with a surge in the daily number of infections during the first few days of the outbreak. In response to this COVID-19 epidemic, temporary static management measures, including city lockdown, mass testing, contact tracing, and isolation of individuals who were infected, were rapidly imposed by the local government on August 10. To block transmission routes of SARS-CoV-2, all individuals with confirmed infections were sent to the designated hospital, and rigorous epidemiological investigations were conducted for each individual to record exposure and contact history. For close contacts of individuals with confirmed infections, comprehensive screening and medical observation were carried out in quarantine settings. Real-time reverse transcription polymerase chain reaction (RT-PCR) testing on a nasopharyngeal or oropharyngeal swab was performed daily for all individuals in Urumqi (ie, citywide mass detection) to actively identify SARS-CoV-2 infections. Since the implementation of these strict control measures, the daily number of infections has gradually decreased.

### SARS-CoV-2 Infections

**Figure 1.**
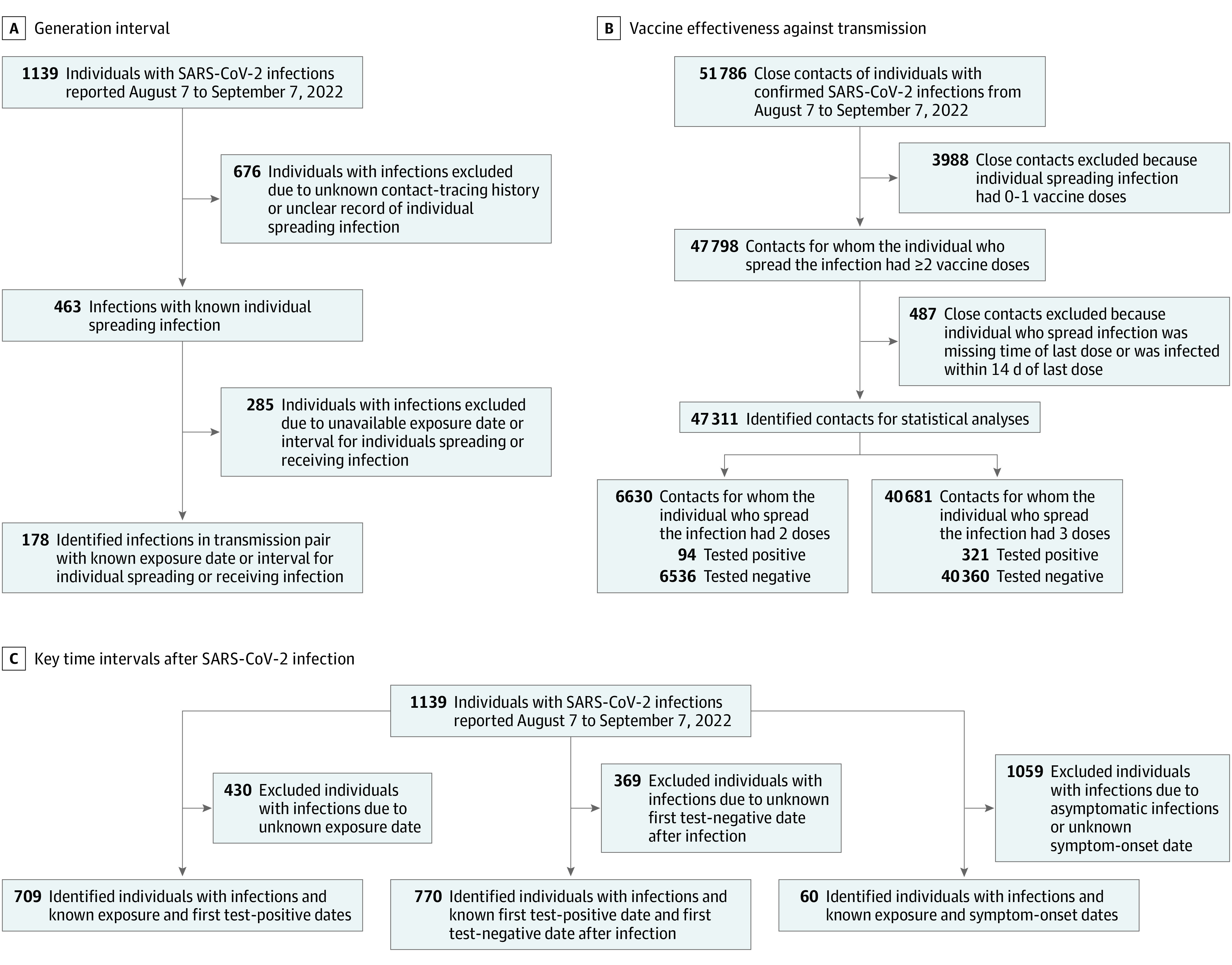
Flowchart of Sample Selection A, The sample selection procedure for transmission pairs that were used for estimating generation interval is presented. B, The sample selection procedure for eligible close contacts who were used for estimating vaccine effectiveness against transmission is presented. C, The sample selection procedure for eligible SARS-CoV-2 infections that were used for estimating the period from exposure to viral shedding, viral shedding period, and incubation period is presented.

We collected epidemiological contact-tracing data of individuals with SARS-CoV-2 Omicron BA.5.2 infections between August 7 and September 7, 2022, in Urumqi from the disease surveillance program of the Xinjiang Uygur Autonomous Region Health Committee. For each individual who was infected, we extracted information on age, sex, exposure history, contact setting (ie, household or nonhousehold), symptom onset date, diagnosis (test-positive) date, serial RT-PCR test outcomes, and vaccination history. We excluded individuals without available patient record information. Contact-tracing data were collected and analyzed as part of an ongoing public health outbreak investigation. See Figure 1 for the flow of data cleaning for several parts of the analysis. Details of the case definition are provided in eAppendix 1 in Supplement 1.

We sequenced complete SARS-CoV-2 genomes for 11 randomly selected individuals infected with BA.5.2 detected within the first week of the local outbreak. Genetic variants of SARS-CoV-2 that seeded infections in this outbreak in Urumqi were classified as the Omicron BA.5.2 sublineage by the PANGO lineage designation. See eAppendix 2 in Supplement 1 for details.

### Close Contacts and Transmission Pairs

We defined close contacts as individuals who had close-contact records with individuals who had laboratory-confirmed SARS-CoV-2 infections. Close contacts included household contacts, social contacts, and close contacts in public spaces.^17^ Based on the exposure history of individuals, we identified epidemiological associations between individuals who were infected and constructed transmission pairs of individuals who spread and received infections. The process of including transmission pairs and individuals who were infected to estimate the distributions of time intervals between key events is shown in Figure 1. Details of close contacts and transmission pairs are provided in eAppendix 1 in Supplement 1.

### Statistical Analysis

We assumed that distributions of time intervals between key events, including the GI, period from exposure to the start of the viral shedding period (ie, a proxy of the latent period), viral shedding period, and incubation period, followed γ distributions and estimated them using a bayesian framework. For the estimation of GI, we also considered possible right-truncation bias in observed transmission pairs; that is, the GI generated by each individual who spread an infection was truncated owing to PHSMs (eg, contact tracing and isolation of individuals who were infected) that could be associated with reduced transmission.^18^ Given that a series of intensive RT-PCR tests was conducted for all individuals who were infected during outbreaks, we used the first date of RT-PCR positivity as a surrogate of the time of viral shedding and considered the last date of positivity before the first negative date as the ending date of viral shedding. This allowed us to calculate the period from exposure to the start of viral shedding and the viral shedding period. The incubation period was directly calculated and fitted to the γ model for transmission pairs with known exposure dates and symptom onset dates. Subgroup analysis was performed for key time intervals by age, contact setting, epidemic period (ie, before or after city lockdown), vaccination status, and symptom status (ie, symptomatic or asymptomatic). Details of statistical approaches for estimating these key time intervals are provided in eAppendix 3 in Supplement 1. We used the Markov chain Monte Carlo method to estimate parameters of γ distributions. The median and 95% credible intervals (CrIs) were obtained from converged posterior distributions of each parameter. In addition, following a previously published theoretical framework,^19,20^ reproduction numbers were estimated and compared before and after city lockdown; see eAppendix 4 in Supplement 1 for details.

Descriptive statistics, including demographic characteristics and contact setting, were generated for local individuals who were infected and their close contacts. Based on contact-tracing data, we described an age-specific contact matrix and a matrix of who acquires infection from whom for the individual who spread the infection, with stratifications of contact setting and epidemic period. See eAppendix 5 in Supplement 1 for details.

We quantified the transmissibility of BA.5.2 variants by the secondary attack rate,^8,16^ which was calculated by dividing the number of infections by the total number of close contacts. Individuals with infections who were associated with zero secondary infections and the close contacts of these individuals were excluded from estimating the secondary attack rate. We also considered the individual reproduction number, defined as the expected number of infections spread by 1 individual with an infection, by calculating the mean number of secondary infections associated with primary infections.^21,22^ The proportion of supercritical transmission is defined by the ratio at which the individual reproduction number is greater than 1,^22,23,24^ and this was measured by the proportion of individuals who spread infections who were associated with more than 1 offspring infection.

Most individuals with SARS-CoV-2 infections in this study (980 of 1139 individuals [86.0%]) received at least 2 doses of vaccine. Thus, we excluded any individual who spread an infection who received fewer than 2 doses of vaccine and their contacts and focused on the VE of 3 doses vs 2 doses (reference level). The odds ratio (OR) of transmission by vaccine status was estimated using logistic regression models. We calculated VE as (1 − OR) × 100% when the OR was 1 or lower or as −(1 − [1 / OR]) × 100% when the OR was greater than 1.^25,26,27^ The VE against transmission was also named as VE against infectivity or infectiousness in some studies and may be interpreted as the reduction in transmission risk from a primary to secondary infection. Potential confounding variables, including sex, age of individuals who spread infections and close contacts, contact setting, vaccine status of close contacts, and calendar date of contact with the individual with the primary infection for each contact, were adjusted in the multivariate model. Adjustment for numerical confounding variables was carried out using the spline function to capture possible nonlinear associations. Subgroup analyses were performed with stratifications, including age, sex, time delay since last dose of vaccine, symptom status, and contact setting. VE estimates were summarized as the median and 95% CrI of converged posterior distributions.

All statistical analyses were performed with R statistical software version 4.1.3 (R Project for Statistical Computing). Bayesian generalized linear regression models were fitted using the package arm.^28^

## Results

**Figure 2.**
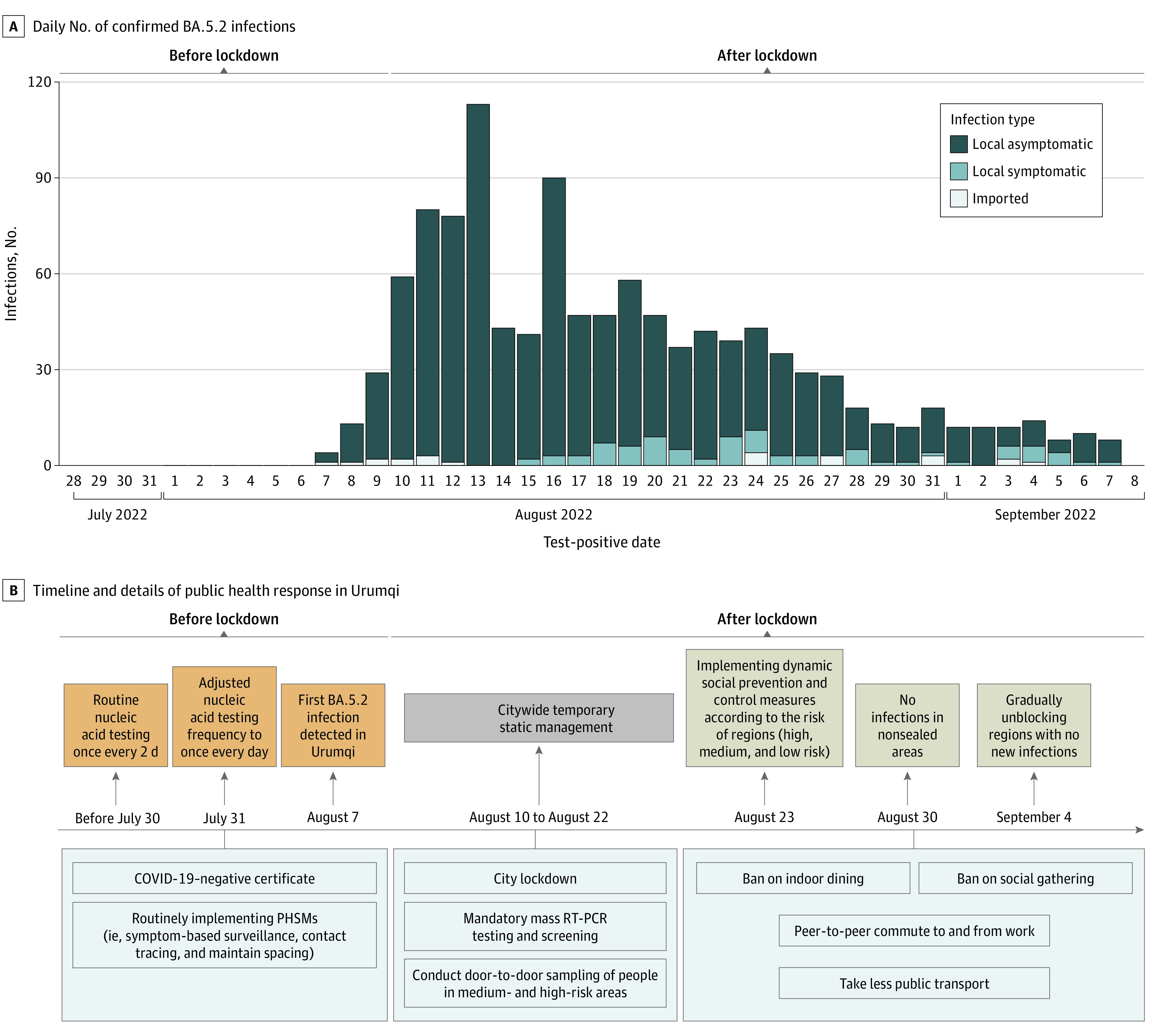
Epidemic Curve of SARS-CoV-2 Infections and Key Public Health Responses A, The daily number of confirmed BA.5.2 infections from August 7 to September 7, 2022, in Urumqi, China, is presented. B, The timeline and details of public health responses in Urumqi stratified by epidemic phase are presented. PHSM indicates public health and social measure; RT-PCR, reverse transcription polymerase chain reaction.

There were 51 323 close contacts who tested negative for COVID-19 (26 299 females [51.2%]; mean [SD] age, 38.4 [16.0] years) of 1139 individuals with confirmed SARS-CoV-2 infections (630 females [55.3%]; mean [SD] age, 37.4 [19.9] years). The daily number of SARS-CoV-2 infections increased from 4 infections on August 7, reaching a peak of 120 infections on August 13 in Urumqi. Starting from August 16, approximately 1 week after city lockdown, the number of daily infections gradually decreased, to 8 infections on September 7. As of September 7, 2022, a total of 1139 individuals infected with BA.5.2 variants were reported in Urumqi, including 23 individuals with imported infections (2.0%), 1033 individuals with local asymptomatic infections (90.7%), and 83 individuals with local symptomatic infections (7.3%) (Figure 2A). After the outbreak of the epidemic, local government rapidly conducted a series of stringent PHSMs to control the spread of COVID-19. The timeline and details of the public health responses in Urumqi are shown in Figure 2B.

Genomic sequencing from 11 individuals with BA.5.2 infections found a total of 62 nonsynonymous mutations^29,30^ (eFigure 1 in Supplement 1). Among these mutations, R203K and G204R may be associated with increased viral RNA replication, load, or virulence.^31^ Among 17 amino acid substitutions found in the receptor binding domain, N501Y and Y505H may be associated with enhanced binding ability of the virus to hACE2 and thus increased viral infectivity^31^ (eAppendix 2 in Supplement 1).

**Figure 3.**
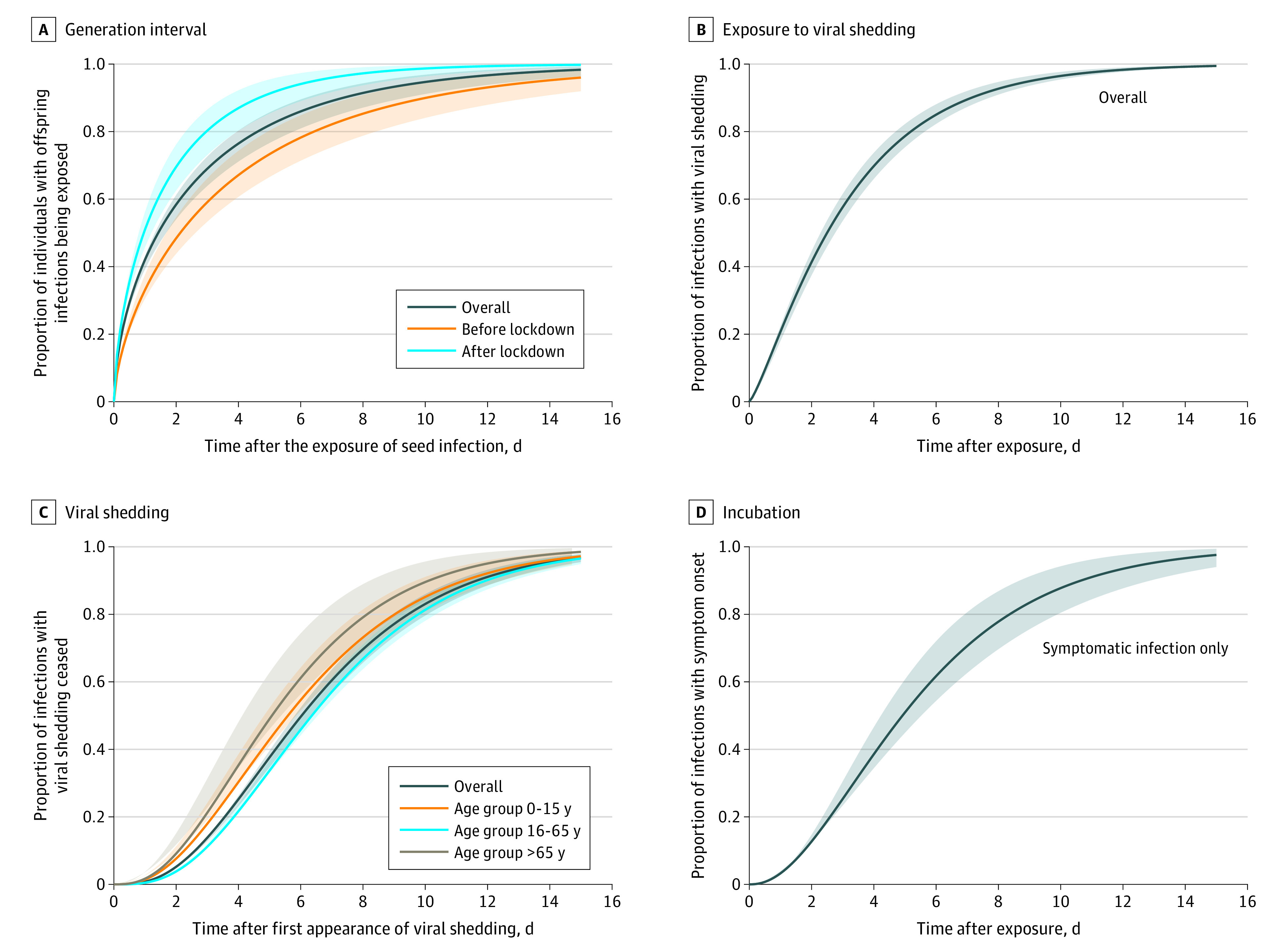
Estimated Cumulative γ Distributions of Key Time Intervals for Omicron BA.5.2 Variant

We analyzed 178 transmission pairs to estimate GI without adjusting for right truncation. We obtained a mean of 2.8 days (95% CrI, 2.4-3.5 days) for GI, with an SD of 3.7 days (95% CrI, 3.0-4.8 days) (Figure 3A). The median GI was estimated at 1.4 days (95% CrI, 1.1-1.9 days), with a 95th percentile of 10.4 days (95% CrI, 8.5-13.0 days). The mean GI estimate was longer in nonhousehold settings than household settings (3.2 days [95% CrI, 2.6-4.0 days] vs 2.3 days [95% CrI, 1.7-3.3 days]) and shorter after the city lockdown compared with the period before lockdown (1.8 days [95% CrI, 1.4-2.4 days] vs 3.9 days [95% CrI, 3.0-5.1 days]). Using estimated GI distributions by epidemic period and estimated exponential growth and decay rates (estimated from the epidemic curve; eAppendix 4 in Supplement 1), we estimated the reproduction number of BA.5.2 to be 3.42 (95% CrI, 3.25-3.45) before the city lock down and 0.46 (95% CrI, 0.45-0.48) after the city lockdown. Additionally, we estimated the GI after correcting for right truncation by using transmission pairs with known isolation dates for the individuals spreading infections. After adjusting for truncation, the mean GI increased to 4.3 days (95% CrI, 2.6-6.9 days) (eTable in Supplement 1). From 60 individuals with symptomatic infections and known exposure times, the incubation period was estimated at 5.7 days (95% CrI, 4.8-6.6 days), with a 95th percentile of 12.8 days (95% CrI, 10.7-15.6 days) (Figure 3D). Individuals with infections and sufficient information on the time of RT–PCR tests were included for the estimation of the period from exposure to the start of the viral shedding period. The mean period from exposure to the start of viral shedding estimated from 709 infections was 3.3 days (95% CrI, 3.0-3.6 days), with a 95th percentile of 8.9 days (95% CrI: 8.1-9.8 days) (Figure 3B). Individuals with asymptomatic infections and individuals who were infected and aged 0 to 15 years had a longer mean period from exposure to viral shedding period (eTable in Supplement 1). For the viral shedding period, we obtained a mean of 6.7 days (95% CrI, 6.4-7.1 days), with a 95th percentile of 13.7 days (95% CrI, 12.7-14.7 days), from 770 individuals with BA.5.2 infections (Figure 3C). Individuals with infections who were asymptomatic and aged 16 to 65 years had longer mean viral shedding periods (eTable in Supplement 1).

Through contact tracing of each individual with an infection, 3040 and 48 746 close contacts of 44 and 725 individuals with symptomatic and asymptomatic infections, respectively, were identified. Of these 51 786 close contacts (including those who tested positive and negative), a total of 1660 and 50 126 contacts were in household and nonhousehold settings, respectively. From 1139 contacts who tested positive, we identified a total of 463 transmission pairs of 236 individuals who spread infections. The age-specific contact and transmission pattern for local infections differed substantially. The most common transmissions occurred from individuals who were aged 5 to 9 years who spread infections to contacts who were aged 10 to 44 years (eFigure 2 in Supplement 1), whereas the frequency of close contacts was the highest between individuals aged 40 to 44 years and 20 to 54 years (eFigure 3 in Supplement 1). Transmission patterns from the age-specific transmission matrix were similar across the epidemic period and contact settings, with more transmissions occurring from younger individuals (ie, those aged 0-15 years) to older individuals (ie, those aged >65 years) (eFigure 2 in Supplement 1). However, the contact pattern differed between household and nonhousehold settings, with a similar frequency of contacts across all age groups in households but a higher number of close contacts between individuals of the same age in the middle age groups (ie, those aged 16-65 years) in nonhousehold settings (eFigure 3 in Supplement 1). Moreover, the contact pattern shifted to the left after the city lockdown; that is, contacts occurred primarily between persons of similar, middle age before lockdown and primarily between younger individuals who spread infections and older individuals after lockdown (eFigure 3 in Supplement 1).

A total of 51 786 close contacts were included in the analysis of the transmissibility of BA.5.2 variants (Table 1). The overall secondary attack rate was 0.9% (95% CrI, 0.8%-1.0%) in all contacts, and the rate was highest in the household setting (14.7%; 95% CrI, 13.0%-16.5%). Among all age groups, individuals who spread infections who were aged 0 to 15 years had a higher secondary attack rate of 2.5% (95% CrI, 1.9%-3.1%); for individuals who spread infections aged older than 65 years, the secondary attack rate was 2.2% (95% CrI, 1.5%-3.0%). Of 1139 index infections that were used to calculate the individual reproduction number (IRN), we found that the mean of IRN was 0.6 (95% CrI, 0.0-0.7), with and SD of 1.33. The IRN remained similar across stratifications, but the supercritical transmission was higher for individuals who spread infections who were older than age 65 years (20.8%; 95% CrI, 10.5%-35.0%).

**Table 1.** SAR, IRN, and SCT Summary

Stratification	Individuals spreading infections, No.^a^	Contacts, No.		Median (95% CrI)		
		Tested positive	Total	SAR, %	IRN^b^	SCT, %^b^
Overall	769	463	51 786	0.9 (0.8-1.0)	0.6 (0.0-0.7)	13.0 (10.7-15.6)
Sex						
Male	313	218	18 711	1.2 (1.0-1.3)	0.7 (0.5-0.9)	14.4 (10.7-18.8)
Female	456	245	33 075	0.7 (0.7-0.8)	0.5 (0.4-0.7)	12.1 (9.2-15.4)
Age, y						
0-15	102	66	2666	2.5 (1.9-3.1)	0.6 (0.4-0.9)	11.8 (6.2-19.7)
16-65	619	362	47 509	0.8 (0.7-0.8)	0.6 (0-0.7)	12.6 (10.1-15.5)
>65	48	35	1611	2.2 (1.5-3.0)	0.7 (0.5-1.1)	20.8 (10.5-35.0)
Symptom status						
Symptomatic	44	25	3040	0.8 (0.5-1.2)	0.6 (0.4-0.8)	11.4 (3.8-24.6)
Asymptomatic	725	438	48 746	0.9 (0.8-1.0)	0.6 (0-0.7)	13.1 (10.7-15.8)
Vaccine doses, No.						
0-1	79	45	3988	1.1 (0.8-1.5)	0.6 (0.4-0.9)	12.7 (6.2-22.1)
2	159	96	6917	1.4 (1.1-1.7)	0.6 (0.4-0.9)	10.1 (5.9-15.8)
3	531	322	40 881	0.8 (0.7-0.9)	0.6 (0-0.7)	13.9 (11.1-17.2)
Contact setting						
Household	515	244	1660	14.7 (13.0-16.5)	0.5 (0.5-0.6)	10.7 (8.2-13.7)
Nonhousehold	714	219	50 126	0.4 (0.5-0.5)	0.3 (0.4-0.6)	5.6 (4.0-7.6)
Epidemic period						
Before lockdown	317	209	36 562	0.6 (0.5-0.7)	0.6 (0.5-0.8)	15.1 (11.4-19.6)
After lockdown	452	115	15 224	0.8 (0.6-0.9)	0.6 (0.5-0.7)	11.5 (8.7-14.8)

Abbreviations: IRN, individual reproduction number; SAR, secondary attack rate; SCT, supercritical transmission.

^a^ This was the sample size of index infections among individuals with number of contacts greater than 0, which was used to calculate SAR.

^b^ For the calculation of IRN and SCT proportions, all 1139 SARS-CoV-2 infections were involved because the number of offspring infections associated with each index infection was known.

In total, 47 311 close contacts of 683 individuals who spread infections were included for estimating VE against the transmission of BA.5.2 (Table 2). Of close contacts, 6630 individuals (14.0%) received 2 doses of vaccine and 40 681 individuals (86.0%) received 3 doses of vaccine. Of individuals who spread infections, 157 individuals (23.0%) had 2 doses of vaccine and 526 individuals (77.0%) had 3 doses of vaccine. The logistic regression model showed that the overall adjusted VE against BA.5.2 transmission for the 3-dose vaccine compared with 2 doses was 28.9% (95% CrI, 7.7%-45.2%). VE against transmission remained considerably high 15 to 90 days after individuals received the third dose of vaccine (48.5%; 95% CrI, 23.9%-61.4%).

**Table 2.** VE of 3-Dose Inactivated Vaccine vs 2-Dose Vaccine

Characteristic	Close contacts, No. (%)				VE (95% CrI)	
	Individuals spreading infection with 2 doses [reference]		Individuals spreading infection with 3 doses		Crude	Adjusted^a^
	Contact tested positive	Contact tested negative	Contact tested positive	Contact tested negative		
Overall	94 (1.4)	6536 (98.6)	321 (0.8)	40 360 (99.2)	44.7 (30.3 to 56.1)	28.9 (7.7 to 45.2)
Sex of individual spreading infection						
Male	54 (1.6)	3666 (98.4)	138 (1.0)	13 439 (99.0)	30.3 (4.3 to 49.2)	28.4 (−4.9 to 51.3)
Female	40 (1.4)	2870 (98.6)	183 (0.7)	26 921 (99.3)	51.2 (31.2 to 65.4)	18.4 (−15.9 to 44.1)
Age of individual spreading infection, y						
0-15	54 (2.5)	2077 (97.5)	0	0	NA^b^	NA^b^
16-65	31 (0.8)	3988 (99.2)	302 (0.8)	39 716 (99.2)	2.2 (−29.3 to 67.5)	3.3 (−32.9 to 29.8)
>65	9 (1.9)	471 (98.1)	19 (2.9)	644 (97.1)	−35.2 (−70.9 to 30.8)	−34.0 (−70.7 to −32.7)
Time since last vaccine dose for individual spreading infection, d						
15-90	3 (1.3)	222 (98.7)	13 (1.1)	1225 (98.9)	21.5 (−64.0 to 77.8)	48.5 (23.9 to 61.4)
91-180	9 (1.0)	862 (99.0)	46 (0.9)	5138 (99.1)	14.3 (−43.1 to 58.2)	13.2 (−44.7 to 58.3)
≥180	82 (1.5)	5452 (98.5)	262 (0.8)	33 997 (99.2)	48.8 (34.2 to 60.1)	19.7 (−4.4 to 38.3)
Symptom status of individual spreading infection						
Symptomatic	8 (0.5)	1668 (99.5)	8 (1.0)	802 (99.0)	−51.9 (−82.0 to 22.3)	−32.0 (−89.4 to 77.1)
Asymptomatic	86 (1.7)	4868 (98.3)	313 (0.8)	39 558 (99.2)	55.2 (43.0 to 64.8)	34.9 (15.1 to 50.1)
Contact setting						
Household	62 (19.1)	267 (80.9)	154 (13.4)	990 (86.6)	33.0 (7.3 to 51.6)	39.1 (12.7 to 57.5)
Nonhousehold	32 (0.5)	6269 (99.5)	167 (0.4)	39 370 (99.6)	16.9 (−17.6 to 43.1)	8.1 (−27.5 to 38.8)

Abbreviations: CrI, credible interval; NA, not applicable; VE, vaccine effectiveness.

^a^ Variables adjusted in the model were sex, age of individuals who spread the infection and got infected, last exposed calendar date of contacts, importation status of individual spreading the infection, contact setting, and vaccine status of individual getting infected.

^b^ These estimates were not calculated owing to insufficient sample size, which led to noninformatively wide 95% CrIs.

## Discussion

SARS-CoV-2 Omicron variants have continually emerged. In this cohort study, we provided a comprehensive analysis of the transmissibility, epidemiological characteristics, and inactivated VE against transmission for the Omicron BA.5.2 subvariant. Our results suggested a higher infectivity for BA.5.2 variants, as indicated by the shorter GI and period from exposure to the start of the viral shedding period compared with other variant of concern and wild type strains.^32,33^ We further found that a booster dose of COVID-19 vaccine was associated with considerable additional protection against BA.5.2 transmission for individuals who received 2 doses. To our knowledge, this is the first study characterizing the transmission dynamics of Omicron BA.5.2 variants, which may potentially contribute to preparations for future outbreak control.

The exposure history of individuals who were infected was obtained by a detailed outbreak investigation, which allowed us to provide robust estimations of the distributions of various key time intervals characterizing transmission events. Our mean estimate of the GI without truncation for BA.5.2 (2.8 days) was shorter than those for the previously circulating Alpha (4.7 days) and Delta variants (5.5 days) obtained from a UK study^32^ but longer than that of the Omicron BA.1 variant (2.4 days) from a Hong Kong study conducted during the initial phase of the outbreak.^11^ Results from the Hong Kong study may be associated with a small sample size and possible sampling bias; that is, a shorter GI was more likely to be sampled during a growing phase of the outbreak.^18,34^ Given that our data were collected from an entire epidemic wave, our results may not have such bias.^34^ Moreover, we obtained right truncation–adjusted GI estimates, which approached the intrinsic GI.^32^ Additionally, we found that the mean GI was shorter in household settings than nonhousehold settings, which could be associated with the depletion of susceptible individuals within households (ie, a decreased potential for observing a longer GI).^32^ Our mean incubation period estimates were longer than previous estimates for BA.1 (3.2-4.6 days)^3^ and BA.2 (4.4 days).^11^ Given that SARS-CoV-2 variants continue to evolve, with changing transmissibility and characteristics, characterizing transmission dynamics of novel variants may be crucial for understanding virus transmission potential and promoting public awareness and preparedness for future outbreaks.

Our mean estimates of the incubation period were greater than that of the period from exposure to the start of the viral shedding period, which suggested a certain proportion of presymptomatic transmission of BA.5.2 variants. In many countries, quarantine and isolation have often been effective control measures during an outbreak. The duration of isolation and quarantine was usually determined by the upper bound of the incubation period distribution (ie, the 95th percentile).^35^ Nonetheless, if RT–PCR tests were conducted regularly during quarantine, the period from exposure to viral shedding period could be more applicable to define the quarantine period, and our findings suggested that testing on the tenth day of quarantine may detect more than 95% of individuals infected before quarantine (95th percentile for the period from exposure to the start of viral shedding: 8.9 days). To date, we are not aware of studies estimating the viral shedding period for Omicron variants, and our mean estimates for BA.5.2 were shorter than those in a previous study on the wild type strain,^36^ possibly associated with the high coverage of vaccination locally.

Although the age-specific contact matrix demonstrated that younger individuals who spread infections had fewer close contacts than older individuals, younger individuals who spread infections were associated with more infections than older individuals who spread infections, as shown in the transmission matrix. Similarly, we observed a higher secondary attack rate for individuals who spread infections who were aged 0 to 15 years than for other age groups. These phenomena may be associated with lower vaccination coverage in younger individuals compared with other age groups in Urumqi and even the entire country. The overall secondary attack rate for BA.5.2 was lower than that for the Delta variant reported by a previous study conducted in Guangzhou, China,^8^ and the secondary attack rate in household setting for BA.5.2 was lower than that for BA.1 and BA.2 from a Danish household study.^4^ The relatively lower secondary attack rates for BA.5.2 could be associated with the higher vaccine coverage in the study population. After city lockdown, the contact matrix shifted to the left, which was possibly associated with more people staying at home so that close contacts among individuals of the same age groups decreased (eg, at the workplace or in social activities).

While there have been many studies evaluating VE against infection and hospitalization,^13,37,38,39,40^ few studies have investigated VE against transmission.^8,41^ The third dose of inactivated vaccine was associated with protection against transmission of the BA.5.2 variant. An increase in VE against transmission of BA.5.2 was found for individuals who received 3 doses of vaccines compared with 2 doses. The VE remained high between 15 and 90 days after the last dose of vaccine, although it decreased dramatically 90 days after the last dose. Although serological analysis was not reported in this study, our results may benefit from laboratory measurement of immune response induced by COVID-19 vaccines (eg, concentration of antibodies and neutralizing titers), which may be needed in further investigations.

### Limitations

Our study has several limitations. First, because our estimated epidemiological characteristics relied on epidemiological contact-tracing data, it is expected that any degree of recall bias and infection underreporting during contact tracing may affect the accuracy of identified transmission pairs and thus bias our GI estimates. Second, because the local population in Urumqi was vaccinated with inactivated vaccine, our result for VE may not be generalizable to other types of vaccine, such as mRNA vaccines. Third, before August 2022, no large-scale COVID-19 outbreak had occurred in Urumqi, and thus we ignored the scenario of reinfection among individuals in this study. Fourth, given that most SARS-CoV-2 infections in this study were asymptomatic (90.7%), some of our findings may not be extendable to individuals who are infected with severe clinical conditions. Fifth, the dominant strain during this COVID-19 outbreak was determined by genetic sequencing results of specimens collected among 11 randomly selected SARS-CoV-2 infections in the first week of the outbreak, and this sampling fraction for genetic sequencing was low. However, we consider that the studied outbreak was seeded by Omicron BA.5 according to background knowledge of circulating strains from public platforms of genetic data and the close population situation during the city lockdown period in Urumqi. Sixth, among the general population in Urumqi, a small proportion of individuals who were infected received 0 or 1 dose of vaccine (86.0% of individuals received at least 2 doses of vaccine), and most of these individuals were ineligible for receiving a COVID-19 vaccine due to existing medical conditions. Thus, we considered individuals who were infected and received 2 doses of inactivated vaccine as the reference group in analyses of VE.

## Conclusions

Findings in this cohort study of individuals with SARS-CoV-2 infections and their close contacts suggested that despite active contact tracing, timely isolation of individuals who were infected, intensive control measures, and high vaccine coverage in Urumqi, Omicron BA.5 variants had high risks of transmission in household settings and younger and older individuals. An evident protective outcome against Omicron BA.5 variant transmission was associated with booster doses of inactivated vaccine (mainly BBIBP-CorV). VE estimates may be important contributions to informing vaccination policy in locations where coverage for a third dose of vaccine remains low or inactivated vaccines were in use. Thus, it is important to assess vaccine performance against emerging genetic variants of SARS-CoV-2 as they evolve regardless of background vaccine coverage.

## Notes

Supplement 1. **eAppendix 1.** Definitions of SARS-CoV-2 Infections, Close Contacts, and Transmission Pairs **eAppendix 2.** Confirmation of Genetic Sequence **eFigure 1.** Key Amino Acid Substitutions in Genetic Sequence Samples From 11 Individuals With COVID-19 **eAppendix 3.** Key Time Intervals of Transmission **eTable.** Estimated Mean, SD, Median, and 95th Percentile of Key Time Intervals for Transmission of SARS-CoV-2 Omicron BA.5.2 Variants **eAppendix 4.** Epidemic Growth and Reproduction Number **eAppendix 5.** Contact Matrix and Mean Number of Offspring Infections by Age of Individuals Spreading and Receiving Infection **eFigure 2.** Who Acquires Infection From Whom Matrix Between Source and Offspring Infections **eFigure 3.** Contact Matrix Between Individuals Who Were Sources of Infections and Their Close Contacts
eReferences.

Supplement 2.
Data Sharing Statement
