# Supplementary material for: Transmission Characteristics and Inactivated Vaccine Effectiveness Against Transmission of SARS-CoV-2 Omicron BA.5 Variants in Urumqi, China
Source: JAMA Netw Open. 2023 Mar 30;6(3):e235755. doi: 10.1001/jamanetworkopen.2023.5755 (PMC10064257; doi:10.1001/jamanetworkopen.2023.5755)
Supplement: Supplement 1. — eAppendix 1. Definitions of SARS-CoV-2 Infections, Close Contacts, and Transmission Pairs eAppendix 2. Confirmation of Genetic Sequence eFigure 1. Key Amino Acid Substitutions in Genetic Sequence Samples From 11 Individuals With COVID-19 eAppendix 3. Key Time Intervals of Transmission eTable. Estimated Mean, SD, Median, and 95th Percentile of Key Time Intervals for Transmission of SARS-CoV-2 Omicron BA.5.2 Variants eAppendix 4. Epidemic Growth and Reproduction Number eAppendix 5. Contact Matrix and Mean Number of Offspring Infections by Age of Individuals Spreading and Receiving Infection eFigure 2. Who Acquires Infection From Whom Matrix Between Source and Offspring Infections eFigure 3. Contact Matrix Between Individuals Who Were Sources of Infections and Their Close Contacts eReferences. [file jamanetwopen-e235755-s001.pdf]

Supplemental Online Content

Wang K, Guo Z, Zeng T, et al. Transmission characteristics and inactivated vaccine effectiveness against transmission of SARS-CoV-2 Omicron BA.5 variants in Urumqi, China. *JAMA Netw Open.* 2023;6(3):e235755. doi:10.1001/jamanetworkopen.2023.5755

eAppendix 1. Definitions of SARS-CoV-2 Infections, Close Contacts, and Transmission Pairs

eAppendix 2. Confirmation of Genetic Sequence

eFigure 1. Key Amino Acid Substitutions in Genetic Sequence Samples From 11 Individuals With COVID-19

eAppendix 3. Key Time Intervals of Transmission

eTable. Estimated Mean, SD, Median, and 95th Percentile of Key Time Intervals for Transmission of SARS-CoV-2 Omicron BA.5.2 Variants

eAppendix 4. Epidemic Growth and Reproduction Number

eAppendix 5. Contact Matrix and Mean Number of Offspring Infections by Age of Individuals Spreading and Receiving Infection

eFigure 2. Who Acquires Infection From Whom Matrix Between Source and Offspring Infections

eFigure 3. Contact Matrix Between Individuals Who Were Sources of Infections and Their Close Contacts

eReferences.

This supplemental material has been provided by the authors to give readers additional information about their work.

eAppendix 1. Definitions of SARS-CoV-2 Infections, Close Contacts, and Transmission Pairs

All COVID-19 cases were defined as individuals who received a positive testing outcome (with Ct value < 40) from real-time reverse transcription polymerase chain reaction (RT-PCR) assay for genetic segments of SARS-CoV-2 strains using ORF1ab gene or N gene detection kit.

The definition of clinical severity after SARS-CoV-2 infection was as follows. For a symptomatic case, it was defined as a case presenting one of the relevant clinical symptoms, including fever, respiratory symptoms, and radiographic evidence of pneumonia. The asymptomatic or mildly symptomatic SARS-CoV-2 infection, which was classified as “asymptomatic” in this study, was defined as a case presenting one of the relevant clinical symptoms for less than 7 days, including fever, or respiratory symptoms, and without a radiographic evidence of pneumonia. Since most of confirmed cases (92.7%) were asymptomatic or mildly symptomatic, which was classified as “asymptomatic” in this study, we avoided to further classify the clinical severity of cases into more detailed levels due to limited samples.

We defined close contacts of a confirmed COVID-19 case as individuals having an epidemiologic link to a COVID-19 case, i.e., this individual has been exposed to another individual with test-positive status for SARS-CoV-2 infection. As a considerable amount of transmission could occur at very early stage after infection [1, 2], individuals who had been exposed to a case within 4 days before the test-positive date of the case would also be counted as close contacts. In this study, we categorized the These individuals who had exposure risk were considered as close contacts (of confirmed cases). Close contacts included, but were not limited to, COVID-19 cases’

- household contacts (i.e., household members regularly living within the same or close space, or relatives who had close contact with case)
- workplace or school contacts (i.e., a work colleague or classmate), and
- community contacts (i.e., healthcare-givers and patients in the same ward, persons sharing a vehicle or restaurant, and community workers having contact with case in public places).

For those contacts who were (eventually) test-positive for COVID-19, we treated these contacts as infectee, and their source case (who were confirmed with COVID-19 in the first place) as infector. We extracted this epidemiologically linked infector and infectee in pairs, and collected their individual-based data for all these transmission pairs.

If it was unclear who was the source case (e.g., more than one infectors were linked to a close contact), this contact would be excluded from analysis. In our study, none of such situation occurs in the dataset.

© 2023 Wang K et al. *JAMA Network Open.*

eAppendix 2. Confirmation of Genetic Sequence

Among the nasopharyngeal or oropharyngeal swabs collected from confirmed COVID cases in the first few days of the outbreak, 11 (out of 38, 28.9%) specimen samples were sent out for whole-genome sequencing. Multiple sequence alignments were performed using MAFFT program. The phylogenetic relationship was explored using maximum likelihood heuristic search and the GTR + CAT nucleotide substitution model in FastTree (version 2.1.11). According to Phylogenetic Assignment of Named Global Outbreak (PANGO) lineage designation, the samples were classified as SARS-CoV-2 Omicron BA.5.2 sub-lineage.

There were a total of 62 amino acid (AA) substitutions in different genetic segments of SARS-CoV-2, including 31 AA substitutions in spike (S) protein, 19 in non-structural proteins (NSPs) ORF1a and ORF1b (S135R, T842I, S538P, A427V, R207C, G1307S, A486V, L3027F, T3090I, S2488F, T3255I, P3395H, P314L, T1050N, S997P, R1315C, I1566V, T2163I and P1727L), 4 in membrane protein (M) (D3N, Q19E, A63T and H125Y), 4 in nucleocapsid (N) (P13 L, R203K, G204R and S413R), 3 in auxiliary proteins ORF3a and ORF9b (T223I, P10S and D16G), and 1 in envelope protein (E) (T9I). The key non-synonymous mutations in these 11 genetic sequence samples were presented in Figure S2.1.

© 2023 Wang K et al. *JAMA Network Open.*

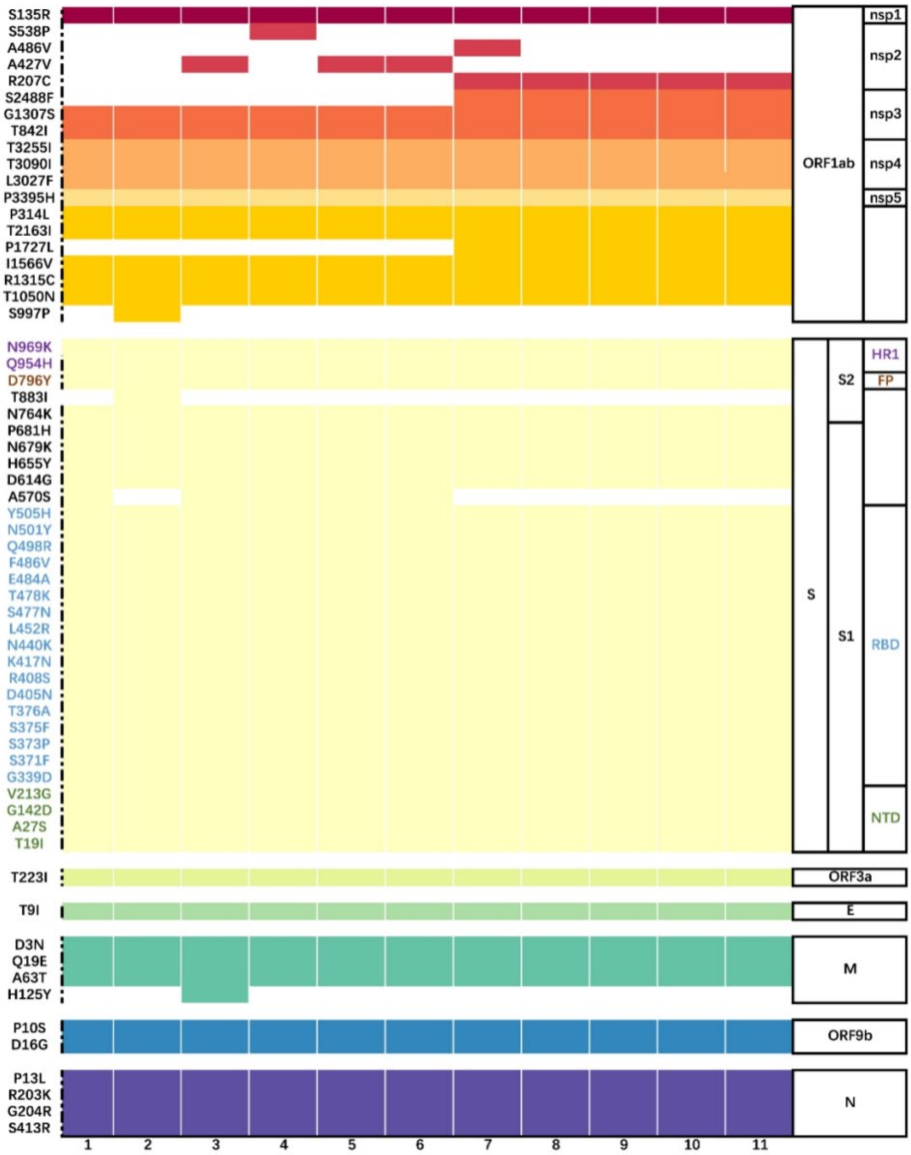

eFigure 1. Key Amino Acid Substitutions in Genetic Sequence Samples From 11 Individuals With COVID-19

Cases labeled as integer from 1 to 11 on the horizontal axis. The colors of text at the left-hand side represented the AA substitutions in NTD (green), RBD (blue), FP (brown), and HR1 (purple).

eAppendix 3. Key Time Intervals of Transmission

S3.1 Generation interval

Generation interval (GI) is defined as the mean duration between time of infection of a secondary infectee and the time of infection of its primary infector [3, 4]. We included transmission pairs with known exposure time for infectors and infectees to estimate the GI. The generation interval together with the reproduction number governs the progression of an epidemic. We denoted G_i as the generation interval (GI) of the ith transmission pair, and we assumed the GI of the Omicron BA.5.2 followed a gamma distribution, denoted by $h(.)$. The GI can change over the course of an outbreak due to sampling bias [5]. In particular, the observed GIs was more likely to be shorter during an exponential growth phase (infectees with more recent exposure to an infector are more likely to be identified) but tend to be longer during the exponential decay phase of the outbreak [5]. In our study, the transmission pairs were collected during a whole epidemic wave that covering both increasing and decreasing phase, and thus our GI estimates would not suffer from such bias. We also considered the right-truncation of the time interval [6-8] that is, the generation interval generated by each infector is truncated due to the PHSMs that could curb the transmission (e.g., contact tracing and case isolation). Thus, the truncation-adjusted distribution function was

$$h_{\text{adjust}}(G_i) = \frac{h(G_i)}{H(T_i)}$$

Here, the $H(.)$ is the cumulative density function of $h(.)$. The T_i is the isolation delay (i.e., the time interval between the isolation and symptom onset) of the infector for the i-th transmission pair. The likelihood functions with and without truncation adjusted are given by:

$$L_{\text{GI}} = \prod_i^n h(G_i)$$

$$L_{\text{GI\_adjust}} = \prod_i^n h_{\text{adjust}}(G_i)$$

S3.2 The period from exposure to viral shedding

The period from exposure to viral shedding was defined as the time interval between exposure and the first appearance of viral shedding, which may be considered a proxy for latent period. To estimate the distribution of the period from exposure to viral shedding, we included cases with known exposure date and RT-PCR test-positive dates. As serial intensive RT-PCR testing were conducted for each confirmed case during the study period, we considered the time that the i-th case start to shed viral genetic particles was bounded by the first test-positive date (denoted by S_{ui}) and the last test-negative date (denoted by S_{li}) of that case. Therefore, the observed period from exposure to viral shedding was interval-censored, which needs to be corrected in the estimation [9]. We excluded cases with a prior to his/her exposure date, which possibly indicating recall bias. We assumed the period from exposure to viral shedding followed a gamma distribution. We denoted E_i as the exposure date of ith case, S_i as the start date of viral shedding, and $w(.)$ as the gamma distribution of the period from exposure to viral shedding. Then the interval-censored likelihood function is constructed as follow:

$$L_{latent} = \prod_i^n \int_{S_{li}}^{S_{ui}} w(S_i - E_i)\,\mathrm{d}S$$

© 2023 Wang K et al. *JAMA Network Open.*

S3.3 Viral shedding period

Confirmed COVID-19 cases with known serial RT-PCR testing date were used to calculate the viral shedding period, which may be considered a proxy for infectious period. Considering the last date for the ith case to shed viral genetic particles was also bounded between the last test-positive date (denoted by R_{li}) and the first test-negative date (denoted by R_{ui}) after infection, the observed viral shedding period was doubly interval-censored [10, 11]. All included cases had an R_{li} later than S_{ui}. We assumed the viral shedding period was gamma distributed, denoted by $u(.)$. Then. we constructed the doubly interval-censored likelihood function as follow:

$$L_{infectious} = \prod_{i}^{n} \int_{S_{li}}^{S_{ui}} \int_{R_{li}}^{R_{ui}} u(R_i - S_i)\, \mathrm{d}R\, \mathrm{d}S$$

S3.4 Incubation period

The incubation period is the duration between the exposure and symptoms onset. We only included symptomatic cases with known exposure date to estimate the incubation period. We denoted V_i as the incubation period of the ith case, and we assumed it followed a gamma distribution, denoted by $k(.)$. The likelihood function is given by:

$$L_{incubation} = \prod_{i}^{n} k(V_i)$$

A graph to show the relationship of GI, incubation period, period from exposure to viral shedding and viral shedding period in Figure S3.1 (see below).

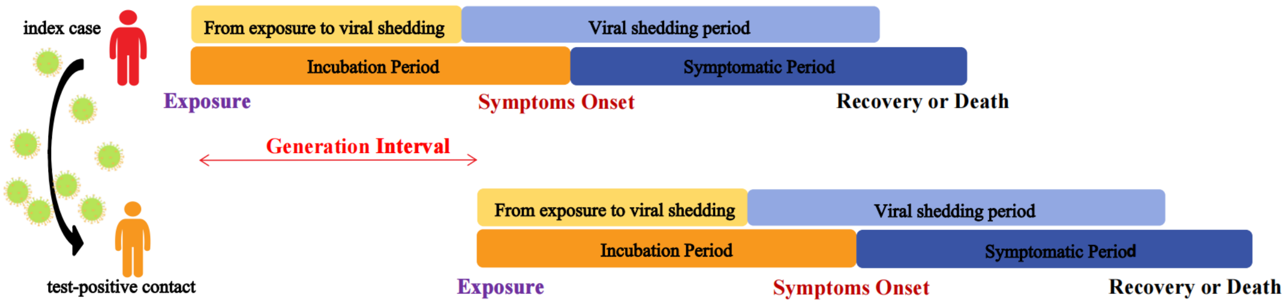

Figure S3.1. The relationship among generation interval (GI), incubation period, period from exposure to viral shedding, and viral shedding period in the context of a typical transmission chain.

S3.5 Parameter estimation

For each key time interval, the parameters of gamma distribution were estimated by using the Metropolis-Hastings algorithm, which is a Markov chain Monte Carlo (MCMC) method, with non-informative prior distributions. The marginal posterior distribution was obtained from 10 000 iterations, among which the first 5 000 samples were discarded as for burn-in. The convergence of each MCMC chain was checked by using the trace plot and Gelman-Rubin-Brooks convergence diagnostic [12]. The median and 95% credible intervals (CrI) were obtained from the converged posterior distributions of each parameter.

S3.6 Supplementary results

The estimations of the key time interval distributions were summarized in Table S3.1.

As for results, we found that the mean GI was shorter in household settings than in non-household settings, which could be attributed to the depletion of susceptible people within household (i.e., a lower potential for observing a longer GI) [13]. The mean GI decreased after the implementation of the lockdown measures, implicitly suggesting that the PHSMs contribute to trimming down the infectiousness profiles of individual cases. An early modelling study indicated that a delay of case isolation was positively associated with the duration of the serial interval [14], a metrics that metric used as a proxy of GI, which means the GI could be shortened on the basis of how quickly fast cases were isolated. Therefore, the GI estimates should also be interpreted in the context of different settings, and our GI estimates would be more generalizable for settings that had the same public health capacity as Urumqi.

© 2023 Wang K et al. *JAMA Network Open.*

eTable. Estimated Mean, SD, Median, and 95th Percentile of Key Time Intervals for Transmission of SARS-CoV-2 Omicron BA.5.2 Variants

All estimates were summarized in "median (95% CrI)" form in the table below.

Time interval	stratification	sample size	Mean	SD	Median	95% percentile
Generation interval	**Overall without truncation**	178	2.8 (2.4, 3.5)	3.7 (3.0, 4.8)	1.4 (1.1, 1.9)	10.4 (8.5, 13.0)
	**Overall with truncation**	79	4.3 (2.6,6.9)	6.7 (4.2,11.1)	1.5 (0.9,2.8)	17.8 (11.1,28.9)
	**contact setting**					
	household	88	2.3 (1.7, 3.3)	3.8 (2.7, 5.3)	0.8 (0.4, 1.3)	9.9 (7.2, 13.9)
	non-household	90	3.2 (2.6, 4.0)	3.4 (2.6, 4.5)	2.2 (1.7, 2.7)	10.0 (8.0, 13.0)
	**epidemic period**					
	before lockdown	83	3.9 (3.0, 5.1)	4.9 (3.7, 6.7)	2.2 (1.5, 3.0)	13.8 (10.6, 18.4)
	after lockdown	95	1.8 (1.4, 2.4)	2.3 (1.7, 3.2)	1.0 (0.7, 1.4)	6.4 (5.0, 8.7)
period from exposure to viral shedding	**overall**	709	3.3 (3.0, 3.6)	2.9 (2.6, 3.1)	2.5 (2.3, 2.8)	8.9 (8.1, 9.8)
	**age group**					
	0-15	130	3.6 (3.0, 4.3)	3.4 (2.7, 4.3)	2.6 (2.2, 3.2)	10.3 (8.5, 12.8)
	16-65	519	3.3 (3.0, 3.5)	2.8 (2.5, 3.1)	2.5 (2.2, 2.7)	8.7 (7.9, 9.8)
	>65	60	3.1 (2.5, 3.8)	2.7 (2.1, 3.6)	2.4 (1.9, 3.0)	8.5 (6.7, 10.8)
	**Symptom status**					
	symptomatic	59	2.8 (2.2, 3.7)	2.5 (2.0, 3.7)	2.1 (1.5, 2.6)	7.8 (6.1, 11.1)
	asymptomatic	650	3.3 (3.1, 3.6)	2.9 (2.5, 3.2)	2.6 (2.3, 2.8)	8.9 (8.1, 10.0)
	**vaccine dose**					
	0-1	92	3.3 (2.8, 4.0)	2.9 (2.3, 3.6)	2.5 (2.0, 3.1)	9.1 (7.3, 11.1)
	2	179	3.5 (3.1, 4.0)	3.1 (2.6, 3.6)	2.7 (2.3, 3.1)	9.5 (8.2, 11.2)
	3	438	3.2 (2.9, 3.5)	2.8 (2.5, 3.2)	2.4 (2.2, 2.7)	8.8 (7.7, 9.8)
viral shedding period	**overall**	770	6.7 (6.4, 7.1)	3.7 (3.4, 4.0)	6.1 (5.7, 6.4)	13.7 (12.7, 14.7)
	**age group**					
	0-15	136	6.3 (5.7, 7.0)	3.7 (3.2, 4.3)	5.6 (4.9, 6.3)	13.3 (11.8, 15.1)
	16-65	574	7.0 (6.6, 7.3)	3.7 (3.4, 4.1)	6.3 (5.9, 6.7)	13.9 (12.9, 15.0)
	>65	60	5.7 (4.9, 6.5)	3.3 (2.7, 4.1)	5.0 (4.2, 5.8)	12.0 (10.1, 14.3)
	**symptom status**					
	symptomatic	53	6.0 (5.1, 7.0)	3.3 (2.6, 4.3)	5.4 (4.6, 6.3)	12.3 (10.2, 14.9)
	asymptomatic	717	6.8 (6.5, 7.2)	3.8 (3.4, 4.1)	6.1 (5.7, 6.5)	14.0 (13.0, 15.1)
	**vaccine dose**					
	0-1	90	6.5 (5.6, 7.4)	4.3 (3.6, 5.4)	5.5 (4.7, 6.4)	14.8 (12.7, 17.9)
	2	188	6.2 (5.7, 6.7)	3.3 (2.9, 3.9)	5.6 (5.1, 6.1)	12.6 (11.3, 14.0)
	3	492	7.0 (6.6, 7.4)	3.6 (3.3, 3.9)	6.4 (6.0, 6.8)	13.8 (12.8, 14.8)
Incubation period	**overall**	60	5.7 (4.8, 6.6)	3.7 (2.9, 4.7)	4.9 (4.0, 5.6)	12.8 (10.7, 15.6)

© 2023 Wang K et al. *JAMA Network Open.*

eAppendix 4. Epidemic Growth and Reproduction Number

The exponential growth (or decay) was frequently used to capture the pattern of epidemic curve [15, 16]. We fitted the observed daily cumulative number of COVID-19 cases denoted by $C(t)$ to an exponential growth model to estimate the growth rate (γ).

Given the estimated growth rate and GI, we estimated the reproduction number (R) by using the formula as per [17]:

$$R = \frac{1}{M(-\gamma)},$$

where the function $M(\cdot)$ was the moment generation function (MGF) of the probability density function (PDF) of GI. For GI following a gamma distribution with parameters shape α and rate β, then the R can be simplified as follows:

$$R = (1 + \frac{\gamma}{\beta})^{\alpha}.$$

© 2023 Wang K et al. *JAMA Network Open.*

eAppendix 5. Contact Matrix and Mean Number of Offspring Infections by Age of Individuals Spreading and Receiving Infection

We relied on contact tracing data to characterize the age-specific contact matrixes and "who acquired infections from whom" (WAIFW) matrixes [18] for the infected individuals and their close contacts. A contact matrix comprised infectors and all of their close contacts (including both test-positive and test-negative individuals), whereas a WAIFW matrix only contains infectors and their test-positive contacts.

All cases were classified into 16 age groups according to their ages (i.e., 0 to 4 years, 5 to 9 years, 10 to 14 years, 15 to 19 years, 20 to 24 years, 25 to 29 years, 30 to 34 years, 35 to 39 years, 40 to 44 years, 45 to 49 years, 50 to 54 years, 55 to 59 years, 60 to 64 years, 65 to 69 years, 70 to 74 years, 75 to 79 years, and 80 years or older), to generate the age-specific contact and WAIFW matrixes. In addition, the age-specific contact and WAIFW matrixes stratified by contact settings and epidemic period (i.e., before and after lock down) were also constructed (see Figure S5.1). The constructed contact matrixes were shown in Figure S5.2.

© 2023 Wang K et al. *JAMA Network Open.*

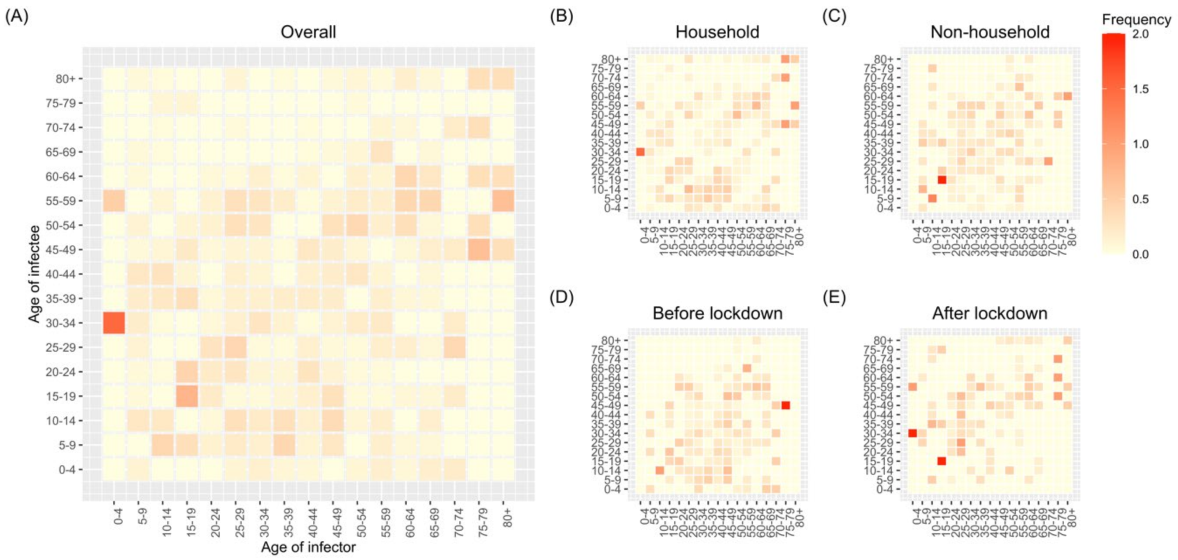

eFigure 2. Who Acquires Infection From Whom Matrix Between Source and Offspring Infections

Stratified by age groups (panel A), and stratified by contact settings including (panel B) household, and (panel C) non-household, and stratified by epidemic periods including the periods (panel D) before city lockdown, and (panel E) after city lockdown.

© 2023 Wang K et al. *JAMA Network Open.*

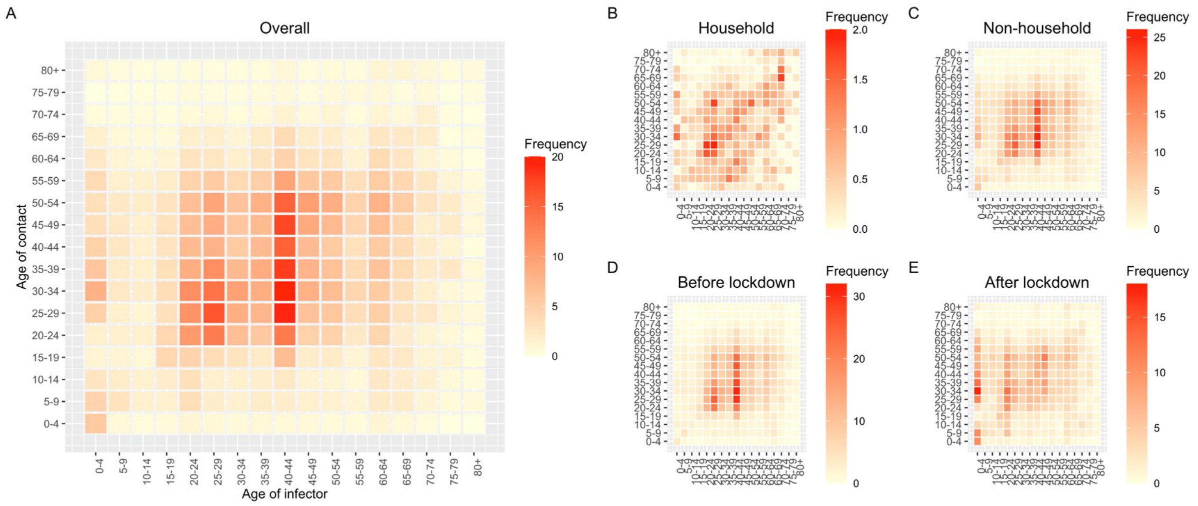

eFigure 3. Contact Matrix Between Individuals Who Were Sources of Infections and Their Close Contacts

Stratified by age groups (panel A), and stratified by contact settings including (panel B) household, and (panel C) non-household, and stratified by epidemic periods including the periods (panel D) before city lockdown, and (panel E) after city lockdown.

© 2023 Wang K et al. *JAMA Network Open.*

eReferences

1. He X, Lau EHY, Wu P, Deng X, Wang J, Hao X, Lau YC, Wong JY, Guan Y, Tan X *et al*: **Temporal dynamics in viral shedding and transmissibility of COVID-19**. *Nature Medicine* 2020, **26**(5).
2. Hu S, Wang W, Wang Y, Litvinova M, Luo K, Ren L, Sun Q, Chen X, Zeng G, Li J *et al*: **Infectivity, susceptibility, and risk factors associated with SARS-CoV-2 transmission under intensive contact tracing in Hunan, China**. *Nat Commun* 2021, **12**(1):1533.
3. Fine PE: **The interval between successive cases of an infectious disease**. *American journal of epidemiology* 2003, **158**(11):1039-1047.
4. Svensson A: **A note on generation times in epidemic models**. *Math Biosci* 2007, **208**(1):300-311.
5. Park SW, Sun KY, Champredon D, Li M, Bolker BM, Earn DJD, Weitz JS, Grenfell BT, Dushoff J: **Forward-looking serial intervals correctly link epidemic growth to reproduction numbers**. *Proc Natl Acad Sci U S A* 2021, **118**(2):12.
6. Zhao S, Gao DZ, Zhuang Z, Chong MKC, Cai YL, Ran JJ, Cao PH, Wang K, Lou YJ, Wang WM *et al*: **Estimating the Serial Interval of the Novel Coronavirus Disease (COVID-19): A Statistical Analysis Using the Public Data in Hong Kong From January 16 to February 15, 2020**. *Front Physics* 2020, **8**:7.
7. Zhao S, Tang BA, Musa SS, Ma SJ, Zhang JY, Zeng MY, Yun QP, Guo W, Zheng YX, Yang ZY *et al*: **Estimating the generation interval and inferring the latent period of COVID-19 from the contact tracing data**. *Epidemics* 2021, **36**:7.
8. Nishiura H, Linton NM, Akhmetzhanov AR: **Serial interval of novel coronavirus (COVID-19) infections**. *Int J Infect Dis* 2020, **93**:284-286.
9. Lau YC, Tsang TK, Kennedy-Shaffer L, Kahn R, Lau EHY, Chen D, Wong JY, Ali ST, Wu P, Cowling BJ: **Joint Estimation of Generation Time and Incubation Period for Coronavirus Disease 2019**. *Journal of Infectious Diseases* 2021, **224**(10):1664-1671.
10. Xin H, Li Y, Wu P, Li Z, Lau EHY, Qin Y, Wang L, Cowling BJ, Tsang TK, Li Z: **Estimating the latent period of coronavirus disease 2019 (COVID-19)**. *Clin Infect Dis* 2022, **74**(9):1678-1681.
11. Reich NG, Lessler J, Cummings DAT, Brookmeyer R: **Estimating incubation period distributions with coarse data**. *Statistics in Medicine* 2009, **28**(22):2769-2784.
12. Gelman A, Carlin JB, Stern HS, Dunson DB, Rubin DB: **Bayesian data analysis, third edition**. *Journal of the American Statistical Association* 2003, **45**(2).
13. Hart WS, Miller E, Andrews NJ, Waight P, Maini PK, Funk S, Thompson RN: **Generation time of the alpha and delta SARS-CoV-2 variants: an epidemiological analysis**. *Lancet Infect Dis* 2022, **22**(5):603-610.
14. Ali ST, Wang L, Lau EHY, Xu XK, Du ZW, Wu Y, Leung GM, Cowling BJ: **Serial interval of SARS-CoV-2 was shortened over time by nonpharmaceutical interventions**. *Science* 2020, **369**(6507):1106-+.
15. Ma J: **Estimating epidemic exponential growth rate and basic reproduction number**. *Infectious Disease Modelling* 2020, **5**:129-141.
16. Zhao S, Musa SS, Lin Q, Ran J, Yang G, Wang W, Lou Y, Yang L, Gao D, He D *et al*: **Estimating the Unreported Number of Novel Coronavirus (2019-nCoV) Cases in China in the First Half of January 2020: A Data-Driven Modelling Analysis of the Early Outbreak**. *Journal of Clinical Medicine* 2020, **9**(2):388.

© 2023 Wang K et al. *JAMA Network Open.*

17. Wallinga J, Lipsitch M: **How generation intervals shape the relationship between growth rates and reproductive numbers**. *Proc Biol Sci* 2007, **274**(1609):599-604.

18. Turner K: **Introduction to Infectious Disease Modelling**, vol. 87; 2010.

© 2023 Wang K et al. *JAMA Network Open.*
