# Supplementary material for: Transmission Characteristics and Inactivated Vaccine Effectiveness Against Transmission of SARS-CoV-2 Omicron BA.5 Variants in Urumqi, China
Source: JAMA Netw Open. 2023 Mar 30;6(3):e235755. doi: 10.1001/jamanetworkopen.2023.5755 (PMC10064257; doi:10.1001/jamanetworkopen.2023.5755)
Supplement: Supplement 2. — Data Sharing Statement [file jamanetwopen-e235755-s002.pdf]

Data Sharing Statement

Wang. Transmission Characteristics and Inactivated Vaccine Effectiveness Against Transmission of SARS-CoV-2 Omicron BA.5 Variants in Urumqi, China. *JAMA Netw Open*. Published online March 30, 2023. doi:10.1001/jamanetworkopen.2023.5755

Data
Data available: No

Additional Information
Explanation for why data not available: The original database containing confidential patient information cannot be made public. The anonymized data that were used in this study is available via reasonable request to the corresponding authors.

© 2023 Wang K et al. *JAMA Network Open*.
